# Comprehensive assessment of COVID-19 case fatality rate and influential factors in Khuzestan Province, Iran: a two-year study

**DOI:** 10.1186/s41043-024-00673-6

**Published:** 2024-11-25

**Authors:** Mohammad-Navid Bastani, Manoochehr Makvandi, Maryam Moradi, Somayeh Biparva Haghighi, Maryam Rostami, Sepideh Nasimzadeh, Homayoun Amiri, Seyed Mohammad Alavi, Mohammad Rashno, Ahmadreza Mohtadi, Farid Yousefi, Abbas Fayezi, Mohammadreza Mirkarimi, Maryam Haddadzadeh Shoushtari, Masoud Zadkarami, Negin Balar, Siamak Mirab Sameii, Mehdi Torabizadeh

**Affiliations:** 1 Virology Department, School of Medicine, Ahvaz Jundishapur University of Medical Sciences, Ahvaz, 15794 – 61357 Iran; 2 Department of Biostatistics and Epidemiology, School of Public Health, Ahvaz Jundishapour University of Medical Sciences, Ahvaz, Iran; 3 Department of English, Faculty of Medicine, Ahvaz Jundishapur University of Medical Sciences, Ahvaz, Iran; 4 Department of Community Medicine, Faculty of Medicine, Ahvaz Jundishapur University of Medical Sciences, Ahvaz, Iran; 5 Deputy of Health, Ahvaz Jundishapur University of Medical Sciences, Ahvaz, Iran; 6 Infectious and Tropical Diseases Research Center, Health Research Institute, Ahvaz Jundishapur University of Medical Sciences, Ahvaz, Iran; 7 Department of Immunology, Faculty of Medicine, Ahvaz Jundishapur University of Medical Sciences, Ahvaz, Iran; 8 Pain Research Center, Imam Khomeini Hospital Research and Development Unit, Ahvaz Jundishapur University of Medical Sciences, Ahvaz, Iran; 9 Department of Infectious Diseases, School of Medicine, Ahvaz Jundishapur University of Medical Sciences, Ahvaz, Iran; 10 Department of Allergy and Clinical Immunology, School of Medicine, Ahvaz Jundishapur University of Medical Sciences, Ahvaz, Iran; 11 Aboozar Hospital, Ahvaz Jundishapur University of Medical Sciences | ajums, Ahvaz, Iran; 12 Air Pollution and Respiratory Diseases Research Center, Ahvaz Jundishapur University of Medical Sciences, Ahvaz, Iran; 13 Department of Pediatrics, Medical School, Ahvaz Jundishapur University of Medical Sciences, Ahvaz, Iran; 14 Reference Health Laboratory, Ministry of Health and Medical Education, Tehran, Tehran, Iran; 15 Clinical Research Development Unit, Golestan Hospital, Ahvaz Jundishapur University of Medical Sciences, Ahvaz, Iran

## Abstract

**Background:**

The emergence of a new pandemic SARS-CoV-2 (COVID-19) resulted in a high mortality rate across the world. This study sought to comprehensively analyze the Case Fatality Rate (CFR) associated with COVID-19 in the Khuzestan province of Iran”. The primary objective was to discern patterns and determinants influencing CFR, shedding light on the evolving impact of the pandemic on morbidity and mortality.

**Methods:**

A retrospective examination was performed on a dataset encompassing confirmed COVID-19 cases and related fatalities in Khuzestan. Data, spanning from December 2020 to April 2022, underwent rigorous statistical analysis. Demographic variables, comorbidities, and temporal trends were scrutinized to identify key factors influencing CFR.

**Results:**

Preliminary findings revealed dynamic shifts in CFR, capturing the nuanced nature of the pandemic over time. Demographic nuances, particularly age and gender, emerged as significant determinants impacting CFR, the reported CFR of COVID-19 in Khuzestan province was 1.79%.

**Conclusion:**

This study contributes critical insights into the CFR landscape of COVID-19 in Khuzestan, providing a foundation for evidence-based decision-making in public health. The identified factors influencing mortality rates offer valuable information for tailoring interventions and optimizing resource allocation. Continuous monitoring and further investigations are recommended to adapt strategies to the evolving nature of the pandemic.

## Introduction

The COVID-19 pandemic, stemming from the emergence of the novel coronavirus SARS-CoV-2 in late 2019, has exerted a profound influence on worldwide health and economies. As the virus continues to spread across countries and continents, understanding the factors that contribute to COVID-19 mortality has become crucial in developing effective strategies to mitigate its impact. One of the most significant measures of the impact of any pandemic is the CFR, which is the number of deaths caused by the disease. In the case of COVID-19, CFRs have been a cause of concern for governments and populations alike.

Iran belonged to the group of nations that experienced a substantial incidence of infections and fatalities amid the COVID-19 pandemic. Specifically, the country recorded more than 7 million confirmed infections and a mortality rate exceeding 146,000 individuals, from January 2019 to October 2024 by the Worldometer website (https://www.worldometers.info/coronavirus/country/iran/). While the majority of COVID-19 cases result in mild symptoms, certain risk factors significantly increase the likelihood of severe illness and death [1].

One of the primary determinants of COVID-19 mortality is advanced age. Elderly individuals, particularly those aged 65 and above, exhibit a significantly elevated susceptibility to severe illness and mortality [2, 3]. Individuals harboring pre-existing medical conditions are likewise at an elevated risk of COVID-19 mortality. Individuals with heart conditions such as coronary artery disease, hypertension, or heart failure are more susceptible to severe COVID-19. The virus can put additional strain on the cardiovascular system, potentially leading to acute cardiac events [4, 5]. People with diabetes have a compromised immune system and may experience more severe COVID-19 symptoms. Elevated blood sugar levels can impair the body's ability to fight off infections [6, 7]. Conditions like chronic obstructive pulmonary disease (COPD) and asthma can result in severe respiratory distress when combined with COVID-19. Patients with these conditions often require hospitalization and oxygen support [8–10]. Obesity is a significant risk factor for severe COVID-19. It can lead to inflammation and respiratory issues, increasing the likelihood of hospitalization and intensive care unit (ICU) admission [11]. Individuals with weakened immune systems, whether due to cancer treatment, organ transplantation, HIV and AIDS, or certain medications, face a higher risk of severe illness [12–14]. Pregnant women, especially in the later stages of pregnancy, are at an increased risk of severe COVID-19. Their changing physiology and the demands on the body can result in more critical illness [15]. Pre-existing medical conditions significantly impact the severity of COVID-19. While the development and distribution of vaccines have provided hope, individuals with underlying health issues must remain vigilant in protecting themselves. The collaboration of healthcare professionals, patients, and the wider community in adhering to safety protocols is vital in reducing the impact of COVID-19 on vulnerable populations [16, 17].

Gender emerges as another noteworthy risk determinant for COVID-19 mortality. Although both males and females can become infected with the virus, research has demonstrated that males exhibit a higher likelihood of encountering severe illness and fatality in comparison to their female counterparts. The precise etiology of this divergence remains incompletely elucidated, but it is conjectured that hormonal, genetic, and behavioral variables could contribute to the discerned distinctions [18].

The cumulative global incidence of COVID-19 cases has escalated to 704,000,000 reported cases, from December 2019 to July 2024, resulting in more than 7 million associated deaths, yielding a case-fatality ratio (CFR) of 0.91%. In parallel, the 22 countries within the Eastern Mediterranean Region (EMR) have documented a collective total of 23,338,462 cases, which accounts for approximately 3.06% of the global caseload, accompanied by 350,704 associated deaths, establishing a CFR of 1.50%. It is noteworthy that the Islamic Republic of Iran has reported the highest number of COVID-19-related deaths regionally, tallying 145,808 fatalities, reflecting a CFR of 1.92%. Furthermore, the total number of administered vaccine doses in the Islamic Republic of Iran has reached 157,785,811 which is the second position in the Eastern Mediterranean Region [19].

**Fig. 1:**
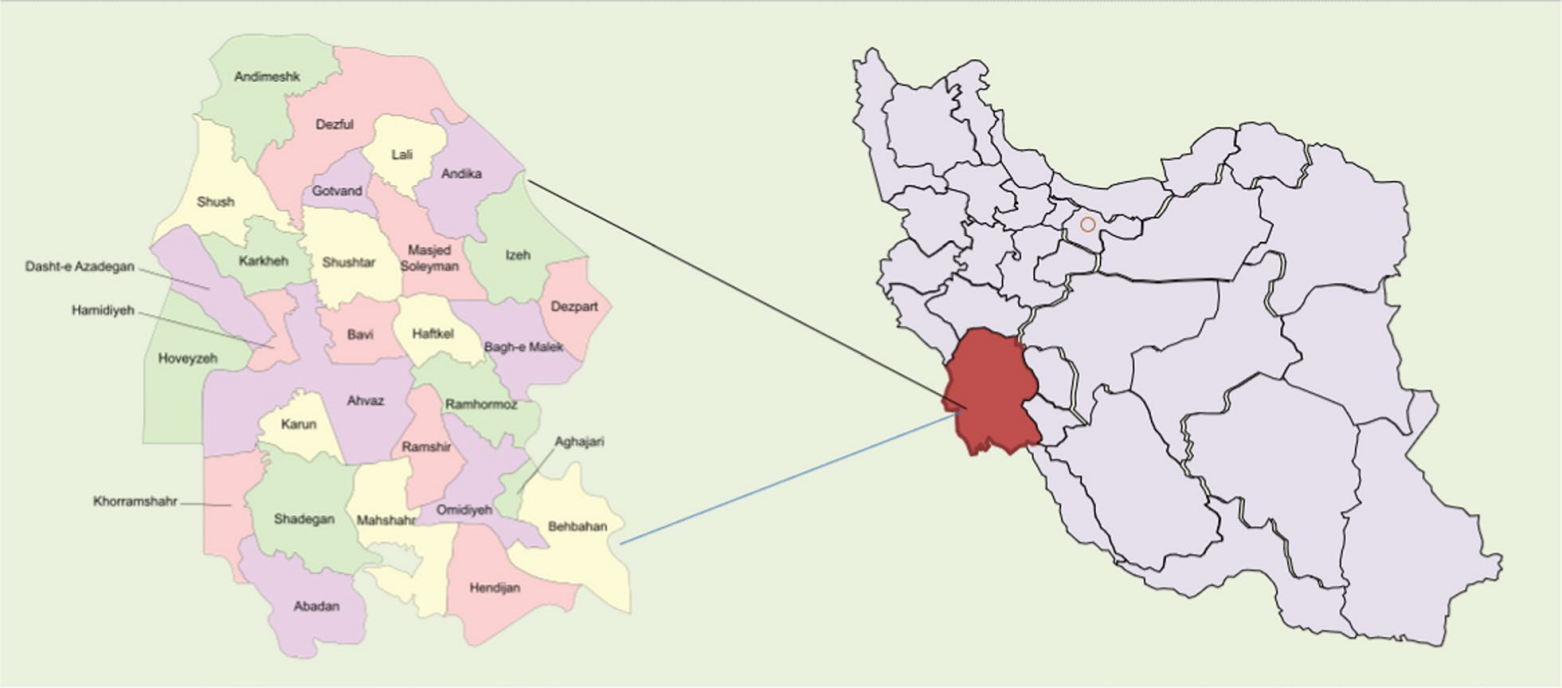
Schematic map of Khuzestan province, Iran

Despite massive worldwide efforts to study COVID-19, critical research gaps remain, notably regarding region-specific variables impacting mortality and the CFR in places with distinct demographic features. The majority of the studies have focused on global or national statistics, frequently neglecting regional differences that might have a significant impact on COVID-19 outcomes. Khuzestan province's excessive death rates are possibly due to a combination of population burdened by pre-existing health issues. However, precise studies on the provincial drivers of CFR, and the interplay of comorbidities in Khuzestan are few (Fig. 1).

This study as Comprehensive Assessment of COVID-19 Case Fatality Rate and Influential Factors in Khuzestan Province, Iran is the first report of CFR in this region of Iran and attempts to fill these gaps by concentrating on Khuzestan province of Iran, an area seriously impacted by the epidemic and with a higher CFR than other regions of Iran. This article attempts to give a better understanding of the mechanisms contributing to COVID-19 mortality by investigating the factors driving CFR in this region, such as comorbidities, and other sociodemographic characteristics. Finally, the study will not only quantify the CFR but also identify the major causes of mortality, allowing for the formulation of more focused public health measures in similar locations confronting healthcare system issues.

## Material and method

The dataset used in this retrospective study was obtained from Jundishapur University of Medical Sciences in Khuzestan through registration in the regional monitoring system of the Covid-19 database and received approval from the Ethics Committee of Jundishapur University of Medical Sciences, Ahvaz, Iran, with the design number CMRC 0024 and ethical number IR.AJUMS.REC.1401.466, wherein the requirement for informed consent was waived. The dataset consisted of records of all patients who received laboratory-confirmed COVID-19 diagnoses as a gold standard through real-time PCR tests conducted on nasopharyngeal and deep nasal swabs according to manufacturer test kit instructions [20]. These patients were registered in the regional COVID-19 registry database in Khuzestan during the period spanning from December 2020 to April 2022. The real-time PCR test kits used in this study were approved by the Iranian Ministry of Health and Education. Reverse transcription qualitative PCR (RT-qPCR) was executed for the N gene and RdRp genes utilizing the one-step RT-qPCR kit (Sansure Biotech, Changsha/Hunan, China), following the guidelines prescribed by the manufacturer. Furthermore, the RNase P gene served as an internal control to assess the accuracy of sample procurement, as well as the RT-qPCR procedure, thereby mitigating the risk of false-negative outcomes. The thermal cycling parameters were established as follows: an initial period of 30 min at 50 °C for reverse transcription followed by 1 min at 95 °C for the activation of PCR initiation, and then 45 cycles of 95 °C for 15 s and 30 s at 60 °C [21].

## Results

**Fig. 2:**
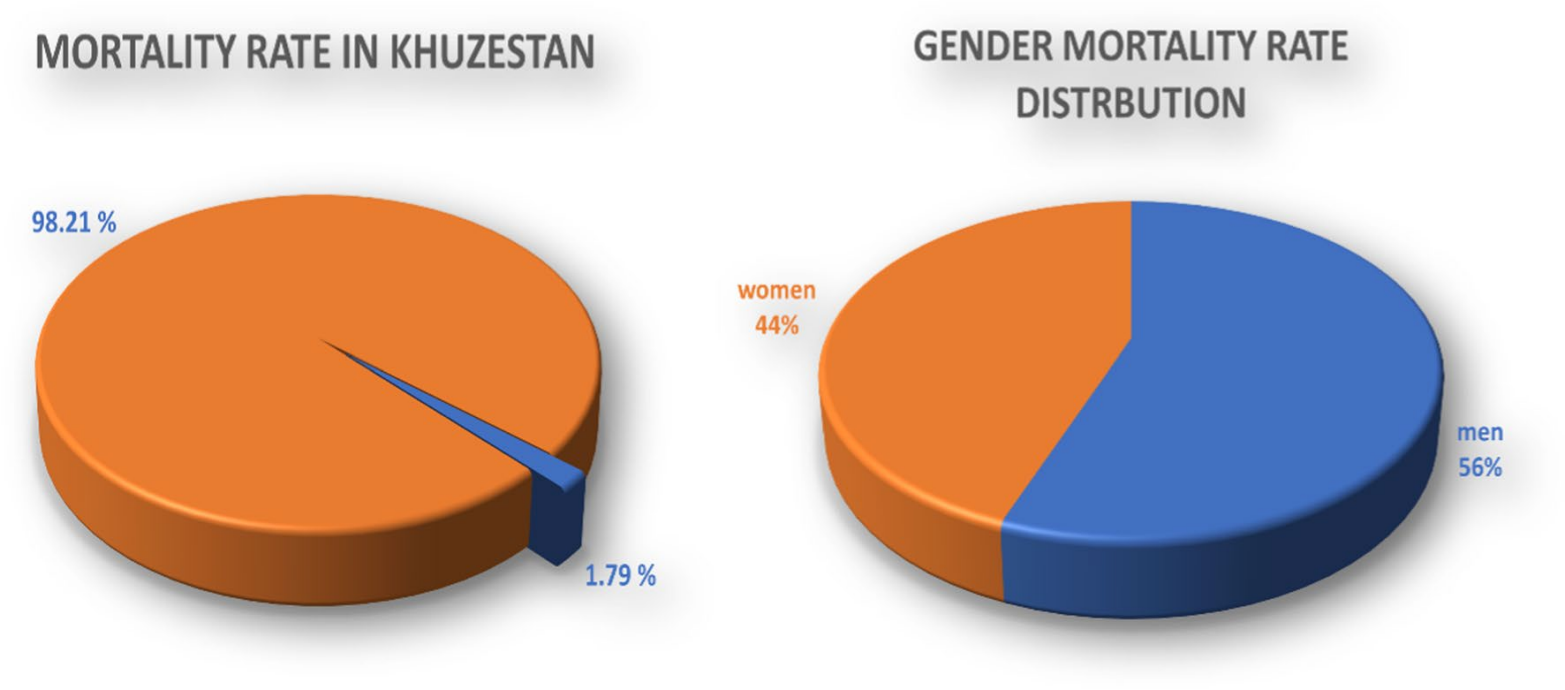
The case fatality rate is documented at 1.79% in the Khuzestan region, with a distribution of 56% among males and 44% among females

Out of 536,742 cases, 295,208 (55%) cases were male and 241,534 (45%) females. Based on the CFR data involving 9,606 individuals in the Khuzestan province, the overall fatality rate among the 536,742 individuals infected with COVID-19 was calculated to be 1.79%. Within this population of COVID-19-infected individuals (536,742), there were 5,414 male fatalities (1.008%) and 4,192 female fatalities (0.781%) (Fig. 2).

Investigation of the CFR attributed to COVID-19 in males and females across various seasons shows the outcomes of the chi-square test indicated a statistically significant disparity between the CFRs of men and women throughout the four seasons of the year, as denoted by the significance level (*P*-value < 0.05). The tabulated values further revealed that during the spring and summer seasons, the CFR among women surpassed that of men, whereas, during the autumn and winter seasons, the CFR among men exceeded that of women (Table 1).

**Table 1:** Case fatality rate in different seasons

			Seasons				Total	Sig
			Spring	Summer	Autumn	Winter		
Sex	Male	Count	1607	2148	970	689	5414	0.000
		% within sex	29.7%	39.7%	17.9%	12.7%	100.0%	
	Female	Count	1323	1783	678	408	4192	
		% within sex	31.6%	42.5%	16.2%	9.7%	100.0%	
Total		Count	2930	3931	1648	1097	9606	
		% within sex	30.5%	40.9%	17.2%	11.4%	100.0%	

The chi-square test findings reveal a statistically significant difference in the CFR between men and women, the tabulated data indicates the incidence of COVID-19-related mortality was consistently greater in males than in women across all age categories. The CFR demonstrated a positive correlation with advancing age, with over 83% of fatalities observed in individuals aged 50 years and above (Table 2).

**Table 2:** Gender and age of COVID-19 mortality cases

Age-sex cross tabulation					
		Sex2		Total	Sig
		Male	Female		
< 10	Count	5	3	8	0.000
	% within age2	62.5%	37.5%	100.0%	
10–20	Count	14	11	25	
	% within age2	56.0%	44.0%	100.0%	
21–30	Count	80	56	136	
	% within age2	58.8%	41.2%	100.0%	
31–40	Count	267	219	486	
	% within age2	54.9%	45.1%	100.0%	
41–50	Count	483	440	923	
	% within age2	52.3%	47.7%	100.0%	
51–60	Count	923	710	1633	
	% within age2	56.5%	43.5%	100.0%	
61–70	Count	1471	1179	2650	
	% within age2	55.5%	44.5%	100.0%	
71–80	Count	1107	924	2031	
	% within age2	54.5%	45.5%	100.0%	
> 80	Count	1064	650	1714	
	% within age2	62.1%	37.9%	100.0%	
Total	Count	5414	4192	9606	
	% within age2	56.4%	43.6%	100.0%	

The following dataset encompassed a comprehensive array of information, which featured medical histories and encompassed conditions such as cardiac disease, chronic pulmonary disease, diabetes, kidney disease, cancer, and other illnesses. In general, although most CFR in Khuzestan is associated with individuals without any health conditions (28.8%), diabetes, hypertension, and cardiovascular diseases comprising over 50% of mortalities, this group occupies the foremost position in the tabulated data. (Table 3).

**Table 3:** comorbidities with COVID-19 mortality

Disease	Abundance	Percent frequency	*P*-value
Absence of disease	2762	28.8	0.000
Diabetes	2730	28.4	
Hypertension	1233	12.8	
Cardiovascular	1031	10.7	
Chronic pulmonary disease	379	3.9	
Kidney	363	3.8	
Stroke	247	2.6	
Obesity	116	1.2	
Chronic neurology	106	1.1	
TSH/T4	78	0.8	
Reduced blood platelets	62	0.6	
Leukemia	59	0.6	
Chronic Hepatic	52	0.5	
Addiction	46	0.5	
IHD	37	0.4	
Breast cancer	36	0.4	
Pregnancy	36	0.4	
Hypothyroidism	34	0.4	
Lung cancer	33	0.3	
Prostate cancer	27	0.3	
Bone marrow cancer	23	0.2	
Clone cancer	16	0.2	
Gastric cancer	13	0.1	
Lymphoma cancer	11	0.1	
Laryngeal cancer	10	0.1	
Bowel cancer	10	0.1	
Skin cancer	10	0.1	
HIV	10	0.1	
Tb	8	0.1	
Hepatitis B	8	0.1	
Lupus	8	0.1	
Uterine and ovarian cancer	6	0.1	
Bladder cancer	6	0.1	
Esophageal cancer	5	0.1	
HCV	5	0.1	

Examining the CFRs attributable to the coronavirus in both genders across diverse diseases, the outcomes of the chi-square test indicate a statistically significant difference in the death rates between men and women, as per the predetermined significance level (*P*-value < 0.05). The corresponding values are presented in Table 4, delineated separately for both men and women.

**Table 4:** COVID-19 case fatality rate contribution in male and female

COVID-19 case fatality rate contribution in male and female					
		Male	Female	Total	Sig
Absence of disease	Count	1571	1191	2762	0.000
	% within disease	56.9%	43.1%	100.0%	
Diabetes	Count	1584	1146	2730	
	% within disease	58.0%	42.0%	100.0%	
Hypertension	Count	625	608	1233	
	% within disease	50.7%	49.3%	100.0%	
Cardiovascular disorders	Count	571	460	1031	
	% within disease	55.4%	44.6%	100.0%	
Chronic pulmonary disorders	Count	213	166	379	
	% within disease	56.2%	43.8%	100.0%	
Kidney disorders	Count	235	128	363	
	% within disease	64.7%	35.3%	100.0%	
Stroke	Count	121	126	247	
	% within disease	49.0%	51.0%	100.0%	
Obesity	Count	76	40	116	
	% within disease	65.5%	34.5%	100.0%	
Chronic neurological disorders	Count	53	53	106	
	% within disease	50.0%	50.0%	100.0%	
TSH/T4	Count	44	34	78	
	% within disease	56.4%	43.6%	100.0%	
Thrombocytopenia	Count	36	26	62	
	% within disease	58.1%	41.9%	100.0%	
Addiction	Count	28	18	46	
	% within disease	60.9%	39.1%	100.0%	
TB	Count	2	6	8	
	% within disease	25.0%	75.0%	100.0%	
Hematopoietic cancers	Count	34	25	59	
	% within disease	57.6%	42.4%	100.0%	
Hepatitis C	Count	1	7	8	
	% within disease	12.5%	87.5%	100.0%	
Chronic Hepatitis	Count	31	21	52	
	% within disease	59.6%	40.4%	100.0%	
Breast cancer	Count	0	36	36	
	% within disease	0%	100.0%	100.0%	
Bone marrow cancer	Count	14	9	23	
	% within disease	60.9%	39.1%	100.0%	
Gastric cancer	Count	10	3	13	
	% within disease	76.9%	23.1%	100.0%	
Pulmonary cancer	Count	20	13	33	
	% within disease	60.6%	39.4%	100.0%	
Laryngeal cancer	Count	5	5	10	
	% within disease	50.0%	50.0%	100.0%	
Esophageal cancer	Count	2	3	5	
	% within disease	40.0%	60.0%	100.0%	
Colon cancer	Count	10	6	16	
	% within disease	62.5%	37.5%	100.0%	
Bowel cancer	Count	8	2	10	
	% within disease	80.0%	20.0%	100.0%	
Uterine and Ovarian Cancer	Count	0	6	6	
	% within disease	0%	100.0%	100.0%	
Bladder cancer	Count	4	2	6	
	% within disease	66.7%	33.3%	100.0%	
lymphoma	Count	5	6	11	
	% within disease	45.5%	54.5%	100.0%	
Pregnancy	Count	0	36	36	
	% within disease	0%	100.0%	100.0%	
Prostate cancer	Count	27	0	27	
	% within disease	100.0%	0%	100.0%	
HIV	Count	7	3	10	
	% within disease	70.0%	30.0%	100.0%	
HCV	Count	1	4	5	
	% within disease	20.0%	80.0%	100.0%	
Lupus	Count	6	2	8	
	% within disease	75.0%	25.0%	100.0%	
Hypothyroidism	Count	24	10	34	
	% within disease	70.6%	29.4%	100.0%	
IHD	Count	21	16	37	
	% within disease	56.8%	43.2%	100.0%	
Total	Count	5414	4192	9606	
	% within disease	56.4%	43.6%	100.0%	

Examining the CFRs attributed to the coronavirus across various age groups and diseases shows the outcomes of the chi-square test revealed statistically significant distinctions. Based on a significance level of *P*-value < 0.05, variations in the CFRs attributable to the coronavirus were observed among different age groups with distinct diseases. The corresponding statistical disparities are delineated in the ensuing table, stratified by age groups. (Table 5).

**Table 5:** Comorbidities and age crosstabulation

		Comorbidities and age crosstabulation										
		0–10	10–20	21–30	31–40	41–50	51–60	61–70	71–80	More than 80	Total	sig
Diabetes	Count	4	8	28	108	255	453	730	610	534	2730	0.000
	% within disease	0.1%	0.3%	1.0%	4.0%	9.3%	16.6%	26.7%	22.3%	19.6%	100.0%	
Cardiovascular disorders	Count	1	2	12	54	69	168	296	236	193	1031	
	% within disease	0.1%	0.2%	1.2%	5.2%	6.7%	16.3%	28.7%	22.9%	18.7%	100.0%	
Chronic neurological disorders	Count	0	0	0	5	8	14	36	24	19	106	
	% within disease	0.0%	0.0%	0.0%	4.7%	7.5%	13.2%	34.0%	22.6%	17.9%	100.0%	
Pulmonary disorders	Count	0	0	4	14	29	61	101	100	70	379	
	% within disease	0.0%	0.0%	1.1%	3.7%	7.7%	16.1%	26.6%	26.4%	18.5%	100.0%	
Kidney disorders	Count	0	2	8	17	34	76	96	78	52	363	
	% within disease	0.0%	0.6%	2.2%	4.7%	9.4%	20.9%	26.4%	21.5%	14.3%	100.0%	
Obesity	Count	0	0	2	3	4	14	25	40	28	116	
	% within disease2	0.0%	0.0%	1.7%	2.6%	3.4%	12.1%	21.6%	34.5%	24.1%	100.0%	
Absence of disease	Count	1	5	37	129	226	420	797	599	548	2762	
	% within disease2	0.0%	0.2%	1.3%	4.7%	8.2%	15.2%	28.9%	21.7%	19.8%	100.0%	
Hypertension	Count	2	7	23	99	180	263	329	179	151	1233	
	% within disease2	0.2%	0.6%	1.9%	8.0%	14.6%	21.3%	26.7%	14.5%	12.2%	100.0%	
TSH/T4	Count	0	1	2	4	7	12	30	12	10	78	
	% within disease2	0.0%	1.3%	2.6%	5.1%	9.0%	15.4%	38.5%	15.4%	12.8%	100.0%	
Thrombocytopenia	Count	0	0	0	2	5	17	20	11	7	62	
	% within disease2	0.0%	0.0%	0.0%	3.2%	8.1%	27.4%	32.3%	17.7%	11.3%	100.0%	
Addiction	Count	0	0	3	5	8	0	8	11	11	46	
	% within disease2	0.0%	0.0%	6.5%	10.9%	17.4%	0.0%	17.4%	23.9%	23.9%	100.0%	
TB	Count	0	0	0	0	0	2	6	0	0	8	
	% within disease2	0.0%	0.0%	0.0%	0.0%	0.0%	25.0%	75.0%	0.0%	0.0%	100.0%	
Hematopoietic cancers	Count	0	0	2	4	5	10	20	10	8	59	
	% within disease2	0.0%	0.0%	3.4%	6.8%	8.5%	16.9%	33.9%	16.9%	13.6%	100.0%	
Hepatitis C	Count	0	0	0	0	3	5	0	0	0	8	
	% within disease2	0.0%	0.0%	0.0%	0.0%	37.5%	62.5%	0.0%	0.0%	0.0%	100.0%	
Stroke	Count	0	0	10	13	37	34	68	52	33	247	
	% within disease2	0.0%	0.0%	4.0%	5.3%	15.0%	13.8%	27.5%	21.1%	13.4%	100.0%	
Chronic Hepatitis	Count	0	0	2	5	10	12	10	8	5	52	
	% within disease2	0.0%	0.0%	3.8%	9.6%	19.2%	23.1%	19.2%	15.4%	9.6%	100.0%	
Breast cancer	Count	0	0	0	6	9	7	6	0	8	36	
	% within disease2	0.0%	0.0%	0.0%	16.7%	25.0%	19.4%	16.7%	0.0%	22.2%	100.0%	
Bone marrow cancer	Count	0	0	0	0	0	10	6	7	0	23	
	% within disease2	0.0%	0.0%	0.0%	0.0%	0.0%	43.5%	26.1%	30.4%	0.0%	100.0%	
Gastric cancer	Count	0	0	1	4	4	2	0	0	2	13	
	% within disease2	0.0%	0.0%	7.7%	30.8%	30.8%	15.4%	0.0%	0.0%	15.4%	100.0%	
Pulmonary cancer	Count	0	0	0	0	3	9	10	10	1	33	
	% within disease2	0.0%	0.0%	0.0%	0.0%	9.1%	27.3%	30.3%	30.3%	3.0%	100.0%	
Laryngeal cancer	Count	0	0	2	2	4	0	0	0	2	10	
	% within disease2	0.0%	0.0%	20.0%	20.0%	40.0%	0.0%	0.0%	0.0%	20.0%	100.0%	
Esophageal cancer	Count	0	0	0	0	0	0	0	1	4	5	
	% within disease2	0.0%	0.0%	0.0%	0.0%	0.0%	0.0%	0.0%	20.0%	80.0%	100.0%	
Colon cancer	Count	0	0	0	0	0	4	9	3	0	16	
	% within disease2	0.0%	0.0%	0.0%	0.0%	0.0%	25.0%	56.3%	18.8%	0.0%	100.0%	
Bowel cancer	Count	0	0	0	0	0	10	0	0	0	10	
	% within disease2	0.0%	0.0%	0.0%	0.0%	0.0%	100.0%	0.0%	0.0%	0.0%	100.0%	
Uterine and ovarian cancer	Count	0	0	0	1	3	2	0	0	0	6	
	% within disease2	0.0%	0.0%	0.0%	16.7%	50.0%	33.3%	0.0%	0.0%	0.0%	100.0%	
Bladder cancer	Count	0	0	0	3	0	0	0	0	3	6	
	% within disease2	0.0%	0.0%	0.0%	50.0%	0.0%	0.0%	0.0%	0.0%	50.0%	100.0%	
Lymphoma	Count	0	0	0	0	0	0	0	6	5	11	
	% within disease2	0.0%	0.0%	0.0%	0.0%	0.0%	0.0%	0.0%	54.5%	45.5%	100.0%	
Pregnancy	Count	0	0	0	3	6	7	11	6	3	36	
	% within disease2	0.0%	0.0%	0.0%	8.3%	16.7%	19.4%	30.6%	16.7%	8.3%	100.0%	
Prostate cancer	Count	0	0	0	0	4	4	9	7	3	27	
	% within disease2	0.0%	0.0%	0.0%	0.0%	14.8%	14.8%	33.3%	25.9%	11.1%	100.0%	
HIV	Count	0	0	0	0	0	0	6	2	2	10	
	% within disease2	0.0%	0.0%	0.0%	0.0%	0.0%	0.0%	60.0%	20.0%	20.0%	100.0%	
HCV	Count	0	0	0	0	0	4	1	0	0	5	
	% within disease	0.0%	0.0%	0.0%	0.0%	0.0%	80.0%	20.0%	0.0%	0.0%	100.0%	
Lupus	Count	0	0	0	2	3	0	0	0	3	8	
	% within disease	0.0%	0.0%	0.0%	25.0%	37.5%	0.0%	0.0%	0.0%	37.5%	100.0%	
Hypothyroidism	Count	0	0	0	1	3	5	11	8	6	34	
	% within disease2	0.0%	0.0%	0.0%	2.9%	8.8%	14.7%	32.4%	23.5%	17.6%	100.0%	
IHD	Count	0	0	0	2	4	8	9	11	3	37	
	% within disease	0.0%	0.0%	0.0%	5.4%	10.8%	21.6%	24.3%	29.7%	8.1%	100.0%	
Total	Count	8	25	136	486	923	1633	2650	2031	1714	9606	
	% within disease	0.1%	0.3%	1.4%	5.1%	9.6%	17.0%	27.6%	21.1%	17.8%	100.0%	

**Fig. 3:**
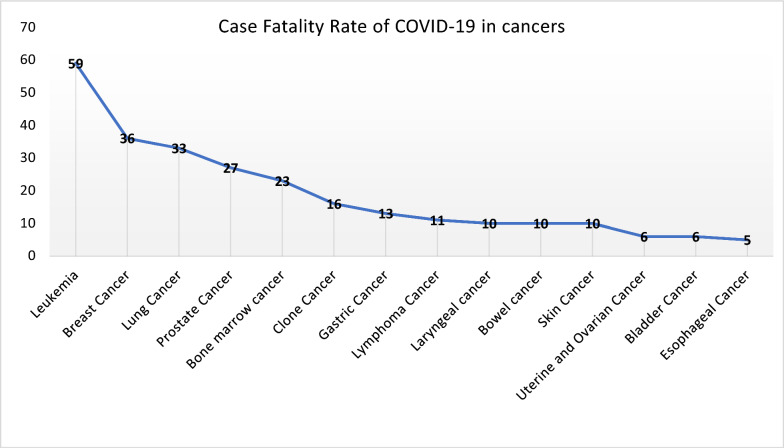
case fatality rate in cancerous patients in Khuzestan

This results revealed a statistically significant increase in the CFR of COVID-19 among individuals with a history of cancer compared to the general population. This suggests that patients with cancer may face a higher risk of severe outcomes if infected with the virus. Stratifying our analysis by cancer type revealed variations in CFR. Patients with certain types of cancers, such as hematologic malignancies, exhibited higher CFR compared to those with other cancer types. Understanding these variations is crucial for tailored clinical management (Fig. 3).

## Discussion

The results of demographic analysis of the individuals who died from COVID-19 in Khuzestan indicate a CFR of 1.79% in this province from December 2020 to April 2022. The CFR in Iran during this time was reported about 1% [22]. The increased outbreak may be attributed to tribal lifestyles and culture and the higher risk of getting COVID-19 in Khuzestan. The mean age of COVID-19 CFR in Khuzestan was (65.67 ± 15.23). In general, the CFR in middle-aged and elderly individuals in the face of this condition is substantially greater than in other ages, which may be attributable to immune system deterioration and exposure to underlying disorders [23, 24]. Statistical analysis revealed the increased CFR in the Khuzestan province during the spring and summer of 2021. The main key reason for this surge in this period was Iran's encounters with the Delta variant of COVID-19. Dealing with restricted vaccination access at the time, this variant led to a record-breaking quantity of fatalities and infections. The Delta strain continued to result in a severe new wave of mortality, and the country faced difficulties in developing herd immunity through natural infection. The Delta variant was associated with elevated transmissibility, with a 60% rise in hospitalization rates compared to the wild type, and elevated viral loads. Furthermore, the Delta variant caused a twofold increase in transmission efficiency compared to the Alpha variant, as well as a 108% boost in hospitalization risk, a 235% increase in ICU admission, and a 133% increase in mortality compared to non-variant concern SARS-CoV-2 strains. By the fall of 2022, the Omicron BA.5 variation had overtaken the Delta variant as the main COVID-19 mutation, resulting in typically milder symptoms [25, 26].

Gender emerges as a critical determinant in COVID-19 mortality, according to this report, males account for 56% of COVID-19 mortality in Khuzestan. As demonstrated by some research examining differences in infection rates and outcomes between men and women. A comprehensive review of COVID-19 mortality data from 49 nations reveals an elevated overall CFR among males [27]. Furthermore, an in-depth look at the severity and mortality of COVID-19 patients indicates that males, regardless of age, are more vulnerable to negative outcomes and mortality [28]. An investigation reveals men attended 1.5 to 2 times more CFR than women among all age categories, making the gender difference in CFR greater than the national difference [29]. In addition, a survey investigating potential explanations for the increased CFR in males with COVID-19 suggests immune system activity, coagulation patterns, prior cardiovascular diseases, and the influence of smoking [30]. Moreover, the expression level of ACE-2 in males was reported higher than in females in the respiratory system, which is associated with more severe signs and symptoms, This might help provide a more receptive microenvironment for SARS-CoV-2 pathogenesis in males [31].

Overall, the increased CFR of males in Khuzestan province is thought to stem from the influence of traditional patriarchal, tribal, and rural cultural norms, wherein the prominence of men is more pronounced throughout the societal framework. It should be noted socioeconomic factors in this province significantly affect COVID-19 mortality. Marginalized populations face barriers to healthcare access, live in crowded conditions, and have limited resources. Those from lower socioeconomic backgrounds may struggle to seek timely medical care and are often employed in essential sectors with higher exposure risks, such as healthcare, transportation, and food services, increasing their vulnerability to the virus [32–34].

Pre-existing medical issues have been linked to an elevated possibility of catastrophic outcomes, such as death, in COVID-19 patients. The top five conditions linked to a surge in COVID-19 mortality are cardiovascular diseases, diabetes, hypertension, respiratory disorders, and chronic kidney diseases, other conditions such as obesity, liver disease, cancer, and neurological disorders have also been associated with increased COVID-19 mortality. The presence of multiple comorbidities has been associated with a greater COVID-19 in-hospital mortality risk [17, 35].

Diabetes is associated with an elevated mortality risk in individuals diagnosed with COVID-19. A comprehensive review and meta-analysis indicate that COVID-19 patients with diabetes mellitus face a significantly higher risk of mortality compared to their counterparts without diabetes mellitus [36]. Moreover, an independent study reported a notable surge in mortality exceeding 30% among individuals with diabetes mellitus during the COVID-19 pandemic [37]. A nationwide survey further revealed a higher CFR in those with type 1 diabetes compared to individuals with type 2 diabetes [38]. It is established that the presence of diabetes enhances the likelihood of experiencing severe manifestations of COVID-19. Despite the precise etiological factors contributing to the escalated CFRs in COVID-19 patients with diabetes remaining elusive, it is postulated that the immunological and inflammatory responses orchestrated by COVID-19, coupled with the existence of comorbidities, collectively contribute to the elevated fatality rates [39]. Research in Mexico discovered that diabetes was related to a hazard ratio for mortality of 1.49 (95% CI 1.47–1.52) in COVID-19 individuals, despite controlling for other variables [40]. Multiple variables may lead to a higher risk: diabetics have impaired phagocytic activity. Decreased neutrophil migration and higher vulnerability to sepsis. Immune dysfunction and Enhanced replication of viruses in high-glucose conditions [41]. Diabetes is the most prevalent pre-existing health condition with high mortality (28.4%) during the COVID-19 pandemic in Khuzestan. The overall incidence of prediabetes and diabetes were 30.8 and 15.3%, respectively in this province. Diabetes prevalence was higher among illiterates, city dwellers, married persons, and smokers. Participants aged 50–65, in addition to those with a BMI of 30 kg/m2 or more, as well as those with hypertension, were more likely to have diabetes [42]. Although the diabetes is second or third comorbidity with high mortality in several studies [43, 44] Regarding the connection between diabetes and other underlying conditions mentioned above, it is reasonable to believe that the CFR of diabetic patients is higher than stated.

One of the most prevalent comorbidities in COVID-19 patients is hypertension, which has a high global incidence, estimated at 30% of the general population, and its frequency increases significantly with age. Furthermore, hypertension frequently interplay with or coexists with comorbidities such as obesity, diabetes, chronic renal disease, and cardiovascular conditions, notably heart failure. Significantly, these comorbidities are recognized risk factors for individuals hospitalized due to COVID-19 [45]. In COVID-19 individuals, the prevalence of hypertension varies from 15 to 50.9% [46, 47]. In this study, the prevalence of hypertension in COVID-19 mortality in Khuzestan was reported 10.8%. A multicenter retrospective investigation of 515 hospitalized COVID-19 patients indicated that the overall mortality was 25.3%, with 73.8% of the deceased patients being hypertensive [48]. Another systematic review and meta-analysis of 23 observational studies comprising 611,522 patients from 5 countries found a 0.5% death rate among COVID-19 patients, with a statistically significant link between hypertension and COVID-19 mortality [49]. A multicenter retrospective cohort research in Wuhan, China, discovered that 40.5% of 1,833 COVID-19 patients had hypertension, and patients with hypertension were more likely to have severe COVID-19 disease and a higher death rate. According to an umbrella study, hypertension was a concomitant condition in 25% of COVID-19 patients, with a 1.79 relative risk for hypertension in COVID-19 mortality [50]. Although hypertension is associated with elevated probabilities of adverse outcomes, encompassing increased infection severity, the occurrence of acute respiratory distress syndrome (ARDS), and elevated CFRs [47, 49, 50], but certain investigations propose that hypertension, when adjusted for other comorbidities in hospitalized COVID-19 patients, may not stand as an independent risk factor for in-hospital mortality [48, 51]; according to a comprehensive update on the relationship between hypertension and COVID-19 mortality, hypertension may not represent the only risk factor associated with an elevated CFR during the COVID-19 pandemic, and an array of other comorbidities and elderly age appears to increase the risk of COVID-19 mortality [52].

The mechanisms underlying hypertension in the pathogenesis of COVID-19 involve a complex interplay of various factors. COVID-19 primarily targets the respiratory system through the angiotensin-converting enzyme 2 (ACE2) receptor, which is highly expressed in the lungs. This interaction may lead to dysregulation of the renin–angiotensin–aldosterone system (RAAS), a key regulator of blood pressure. Accumulation of angiotensin II as a vaso-constrictor leads to elevation of blood pressure and also promotes inflammation, oxidative stress, and endothelial dysfunction, further contributing to hypertension and worsening COVID-19 outcomes [53]. since hypertension serves as a foundational element in other medical conditions acknowledged as risk factors for COVID-19, it appears that its associated CFR may exceed the given estimates.

Cardiovascular diseases (CVD) remained the top reason for mortality in Iran, accounting for a million disability-adjusted life years (DALYs) [54]. CVD contributes significantly to COVID-19 CFRs. The association between CVD and COVID-19 is multifaceted, with both direct and indirect effects of the pandemic on cardiovascular health. COVID-19 can directly impact the cardiovascular system, inducing myocarditis, cardiogenic shock, cardiac arrhythmia pulmonary embolism, venous embolism, sudden heart failure, and myocardial infarction. The virus can also exacerbate pre-existing heart disease, which is usually exacerbated by cardiovascular issues. Patients admitted to an ICU with cardiac failure plus COVID-19 had a significantly greater mortality risk of up to 75% [5]. Between March 2020 and June 2021, a cumulative total of 600,241 fatalities associated with COVID-19 were documented in the United States, among these cardiovascular disorders the rate of mortality was 32.7% [55]. In this study, the CFR of cardiovascular disease was reported 10.7%. According to a meta-analysis investigation, people with underlying cardiovascular disease were 3.44 times more likely to have severe or fatal COVID-19 [56]. Due to the multiple nature of cardiovascular diseases, such as obesity, diabetes, lung issues, it is thought that the CFR should be greater.

The CFR in COVID-19 patients is significantly impacted by pulmonary illnesses. COVID-19 has a 25-fold higher mortality risk for individuals with chronic lung disorders such as chronic obstructive pulmonary disease (COPD), lung cancer, and interstitial lung diseases (ILDs) [57]. In this study, the CFR of pulmonary disorders including lung cancer was reported as 4.2%. The CFR (CFR) of COVID-19 varies among hospitalized adult patients in different countries, ranging between 4 and 11% [22]. The relationship between pre-existing lung disorders and COVID-19 severity emphasizes the significance of customized therapies and public health policies. Patients diagnosed with chronic obstructive pulmonary disease (COPD) have been observed in 50–52.3% of the total COVID-19 cases admitted to the intensive care unit (ICU), resulting in elevated mortality rates within this cohort [58, 59]. While the exact CFR for COPD patients is not explicitly outlined in the search findings, the substantial proportion of ICU admissions and the associated increased mortality strongly imply a significantly heightened CFR in this particular demographic. Regrettably, the search outcomes lack specific CFR values for individuals with alternative forms of pulmonary disorders affected by COVID-19. Nonetheless, considering the heightened susceptibility and severity of COVID-19 in individuals with chronic lung diseases, it is reasonable to deduce that the CFR in these patients is likely higher than that observed in the general population. A multifaceted approach that considers the unique challenges faced by individuals with pulmonary diseases is essential for reducing the overall CFR and safeguarding the health of vulnerable populations.

Stroke, a cerebrovascular event, has surfaced as a critical role in the prognosis of COVID-19 patients. Recent studies suggest that individuals with a history of stroke may face a higher risk of severe illness and a higher CFR when infected. The proposed pathogenesis for stroke in patients with COVID-19 involves an interplay of vascular risk factors and immune responses to the SARS-CoV-2 virus. The pathways of stroke in COVID-19 include a hypercoagulable state, vasculitis, and cardiomyopathy [60]. In this study, the CFR of stroke was reported as 2.6% of all mortality. According to a review and meta-analysis, there exists a notable association between stroke and increased COVID-19 mortality, as indicated by a pooled effect of 1.30 [61]. The risk of experiencing an ischemic stroke among hospitalized COVID-19 patients was observed to be 1.8%, surpassing the incidence rate documented in individuals with influenza [62]. According to the World Health Organization, the likelihood of ischemic stroke in COVID-19 patients stands at approximately 5%, resulting in a CFR 3.2–7.8 times higher (approximately 38%) than that observed in non-COVID-19 stroke patients. Additionally, it is imperative to recognize that the severity of COVID-19 infection significantly influences the outcomes of stroke patients, contributing to an increased fatality rate [63].

Subsequently, obesity and chronic neurological diseases emerged as contributory factors to mortality in Khuzestan province, with CFRs of 1.2% and 1.1%, respectively. An increased body mass index (BMI) elevates the chance of serious disease, hospitalization, admission to ICU, and death. In COVID-19 individuals, obesity is substantially related to more severity and mortality. As a result, it is advised that obesity or its surrogate body mass index be included in prognostic ratings and that recommendations for patient care management be improved. Neurological symptoms have been reported in 35–50% of COVID-19 cases [64]. Mental and neurological disorders have been associated with COVID-19 infection, severity, and mortality [65].

The CFR of COVID-19 in cancerous patients varies according to the kind of malignancy. Based on the data compiled in this investigation, leukemia, lung, breast, prostate, and bone marrow cancers emerge as the primary five malignancies associated with an elevated CFR in COVID-19 with approximately 2.8% within the Khuzestan region. An investigation reveals a higher incidence of cancer in men within the Khuzestan region. Nevertheless, the findings of this study highlight that the most commonly occurring cancers in men include skin, prostate, and lung cancers, while in women, they consist of breast, skin, and hematopoietic malignancies. Furthermore, the mortality rate of cancers in men is higher than women [66]. In the past few years, Khuzestan has witnessed a significant increase in leukemia incidence and death [67]. Leukemia is the most prevalent cancer with high mortality of 22% among cancerous patients with COVID-19 in this study. Statewide population-based research in Taiwan showed a 1.9% CFR for COVID-19 individuals with chronic myeloid leukemia (CML) [68]. An investigation conducted in New York discovered a CFR of 4.9% among 285 individuals diagnosed with cancer and COVID-19 at the same time [69]. A comparison of cancer and non-cancer patients revealed that cancer patients had a CFR of 22.2% within 21 days after COVID-19 diagnosis, over the 15.6% CFR reported in non-cancer patients [70]. It is critical to keep in mind that these CFRs vary depending on parameters such as age, comorbidities, cancer subtype, and stage, as well as the study's chronological and geographical setting. Individuals with both cancer and COVID-19 have an increased risk of mortality, necessitating the deployment of suitable preventative interventions to reduce their risk.

## Conclusion

By July 2022, Iran reported over 7.2 million confirmed COVID-19 cases and 141,350 fatalities, while administering at least 149,957,751 vaccine doses. By November 2022, vaccination coverage reached 69.62% of the population, following the launch of the COVID-19 immunization program in February 2021. Iranian domestic COVID-19 vaccines, COVIran Barekat, PastoCovac, and SpikoGen, were launched to the general public in June, July, and October of 2021, respectively [71]. The attenuation of the delta wave of COVID-19, coupled with the initiation of vaccination campaigns in Iran, appears to have resulted in a decline in the CFR.

Future avenues for comprehending the COVID-19 CFR in Khuzestan, Iran, encompass an enhancement of healthcare resource accessibility and a deeper exploration of the specific factors contributing to the elevated CFR within the region. Given the higher prevalence of comorbidities in the local population, endeavors to ameliorate these conditions may also prove instrumental in mitigating COVID-19-related mortality. Moreover, further investigation is imperative to gain a more profound insight into how sociocultural elements may exert influence on the transmission and severity of the disease in this geographic area. Collaborative initiatives involving healthcare professionals, governmental authorities, and community stakeholders will be pivotal in addressing the intricate public health challenges and the burden of COVID-19 in Khuzestan, Iran.

### Study limitations

Fluctuations in resource availability, hospital capacities, and treatment protocols over time may have influenced patient survival rates, thereby affecting the observed case fatality rate (CFR) trends. Additionally, the study did not systematically account for the impact of different viral strains on CFRs, and the absence of variant-specific data limits the assessment of how these strains may have affected mortality rates. A further significant limitation is the lack of detailed individual-level data on key factors, such as vaccination status, type of vaccine administered, and access to treatment interventions. These variables could have substantially influenced outcomes and impacted the interpretation of CFR trends, particularly when comparing different study periods. Consequently, these limitations introduce potential biases and external influences that must be considered when interpreting the study’s findings.

## Data Availability

We do not analyse or generate any datasets, because our work proceeds within a theoretical and mathematical approach.
